# Serum Micronutrient Status of Haart-Naïve, HIV Infected Children in South Western Nigeria: A Case Controlled Study

**DOI:** 10.1155/2014/351043

**Published:** 2014-08-11

**Authors:** H. C. Anyabolu, E. A. Adejuyigbe, O. O. Adeodu

**Affiliations:** Department of Paediatrics and Child Health, Obafemi Awolowo University, Private Mail Bag 13, ILE-IFE, Nigeria

## Abstract

*Background*. Though micronutrients are vital in the pathogenesis of human immunodeficiency virus infection, most studies have been conducted in adults. Knowledge of the status of key micronutrients in HIV infected African children will indicate if supplementation may be beneficial to these children living in this resource-poor region. *Objectives*. We sought to determine the micronutrient status and associated factors of HAART-naïve HIV infected children and compare them with those of the HIV negative controls. *Methods*. We enrolled 70 apparently stable HAART naïve HIV infected children. Seventy age and sex matched HIV negative children were equally enrolled as the controls. Their social class, anthropometry, clinical stage, CD4 counts, serum zinc, selenium, and vitamin C were determined. *Results*. The prevalence of zinc, selenium, and vitamin C deficiency in HIV infected subjects was 77.1%, 71.4%, and 70.0%, respectively, as compared to 44.3%, 18.6%, and 15.7% in HIV negative controls. Among the HIV infected subjects, 58.6% were deficient in the three micronutrients. Micronutrient status was related to the weight, clinical, and immunological stages but not BMI or social class. *Conclusion*. Deficiency of these key micronutrients is widely prevalent in HAART naïve HIV infected children irrespective of social class. This suggests that supplementation trial studies may be indicated in this population.

## 1. Introduction

With an estimated 321,000 children living with the human immunodeficiency virus (HIV) in Nigeria as at 2010, it is currently established that the country has the largest burden of paediatric HIV infection in the world [1]. HIV infection has been associated with low selenium status in Western nations [2] and Nigeria [3]. The utilization of host selenium by HIV to form selenoproteins essential for viral replication has been postulated as contributory to the low selenium level [2]. In a prospective cohort study of 670 children born to HIV-infected women, low plasma selenium levels were associated with an increased risk of mortality [4].

Zinc has a specific role as an antioxidant and an immune modulator and possibly has a direct antiviral effect [5]. It is a micronutrient of key interest in efforts at curtailing the morbidity and mortality of HIV infection [6]. Low serum zinc has been reported in Ugandan HIV infected children [7]. At present, the zinc status of HAART naïve HIV infected children in Nigeria is unknown.

Vitamin C is the major water-soluble antioxidant and acts as the first defence against the tissue-damaging reactive oxygen species produced during infections [8]. Prevalent vitamin C deficiency has been observed in an earlier study on HIV infected adolescents [9]. Vitamin C has therefore become a key component of various micronutrient supplementation studies in HIV infected children [10–12].

Both selenium [13] and zinc [14] are bound to albumin for their transportation within the plasma. Hypoalbuminemia therefore causes low serum levels of selenium and zinc, though the total body content may still be normal. In malnourished HIV infected patients, their serum albumin level should be taken into account and controlled for before conclusions are drawn regarding the serum selenium and zinc concentrations.

A recent Cochrane review [15] has identified three micronutrients (selenium, zinc, and vitamin D) as having a huge potential for reducing HIV related morbidity and mortality. Formulation of a supplementation programme relevant to our population will require knowledge of the micronutrient status of HIV infected children in our population as micronutrient needs vary widely across diverse regional settings [16]. Most of the pre-HAART micronutrient studies in the literature relate to adults. The few that were done in children either lacked HIV negative controls [6, 17] or had a relatively small sample size [18]. Furthermore, there is a lack of data on micronutrient status of HIV infected children in Nigeria. A case-controlled study in Nigeria on micronutrient levels of HIV infected children will therefore be of great relevance.

The objective of the study was to determine the serum levels of zinc, selenium, and vitamin C in HIV infected children in Obafemi Awolowo University Teaching Hospitals Complex (OAUTHC), Ile-Ife, as well as the relationship to disease severity and nutritional status.

## 2. Materials and Methods

The study was conducted at the paediatric consultant outpatient clinic of the Ife Hospital Unit (IHU) of the OAUTHC Ile-Ife, Osun State, Nigeria. The IHU is a three hundred bedded tertiary health facility located about a hundred and sixty kilometres northeast of Lagos.

Although, the paediatric HIV clinic started in 1997, there were no free drugs for treatment until in 2005 when OAUTHC was included in the National Antiretroviral Treatment Rollout Programme. The hospital also receives referrals and transfers of patients from all the southwestern states and beyond. Over three hundred HIV infected and exposed children are seen in the clinics monthly. The hospital serves as the first port of call for most of the referred, transferred, and newly identified HIV infected children. HIV negative general paediatric cases are seen simultaneously as well. The latter group was the major source of the study controls aside from other paediatric related clinics such as paediatric orthopaedic and paediatric surgery clinics.

The study was a hospital-based, cross-sectional, descriptive, and case control study. All consecutive newly identified HIV infected children over 18 months of age were eligible for recruitment into the study. Patients were excluded from the study when there was (a) lack of consent from the child's care giver, (b) any on-going febrile illness, diarrhoea, or history suggestive of tuberculosis or liver disease such as hepatitis, and (c) on-going supplement usage at the time of the study or within the month preceding it, and (d) when subjects were on antiretroviral therapy [20, 21].

The study commenced in August 2007 and ended in January 2009. A total of 70 HIV infected children were recruited. Each of them was age and sex matched with a HIV negative apparently healthy child as the control. Thus a total number of 140 children (subjects and controls) were recruited into the study.

An ethical clearance certificate was obtained from the Research and Ethical Committee of the OAUTHC before the study was commenced.

### 2.1. Data Collection

All recruited subjects and controls were interviewed by the investigator according to the study proforma to determine their age, tribe, and address. The socioeconomic status was also ascertained according to Oyedeji social classification method [19]. The World Health Organization revised clinical staging was used to stage each HIV infected subject [22]. Weights and heights were measured according to standard methods [23]. Each measurement was taken twice by the investigators assisted by resident doctors, after which the mean was recorded. Pretest and posttest counselling were offered to all of the patients in compliance with the WHO guidelines on provider-initiated testing and counselling (PITC) [24]. HIV screening was done in the haematology laboratory of the OAUTHC by rapid testing using the parallel method according to the national algorithm of HIV testing in use at that time [25]. The two test kits were Determine^R^ HIV-1/2 (Abbot Laboratories, Japan) and Chembio HIV 1/2 STAT-PAK, (Chembio Diagnostic System Inc. New York, USA). Afterwards, subjects as well as controls were offered posttest counselling and then recruited into the study if they granted consent. Subjects and controls were nonfasting and the blood samples were collected in the morning (i.e. before 12 noon). This was then shared as follows: four millilitres dispensed into plain specimen tube while the two millilitres was emptied into ethylenediaminetetraacetic acid (EDTA) bottles. The samples in the plain tube were left still in a cool corner, protected from light, to allow for sufficient clotting and retraction. This process took less than four hours after which they were then spun with a centrifuge at 3,000 revolutions per minute for 10 minutes. The serum was thereafter separated with a bulb pipette. Two millilitres of serum was usually derived. Half millilitre was sent to the OAUTHC Chemical Pathology Laboratory for albumin while the rest was immediately kept in a laboratory freezer at −20°C until analysis for the micronutrients.

### 2.2. Sample Analysis

Zinc and selenium were analysed at the Central Science Laboratory of Obafemi Awolowo University, Ile-Ife, with a flame atomic absorption spectrophotometer (model 210) manufactured by Buck Scientific Corporation, Connecticut, USA. The Nigerian Food Consumption Survey of 2003 [26] used 80 *μ*g/dL as the lowest limit of normal for zinc. Values lower than 80 *μ*g/dL were taken to indicate zinc deficiency in this study. For serum selenium, values less than 85 mg/L are generally defined as deficient [27]. Vitamin C concentration was also measured by spectrophotometry using 2, 4-dinitrophenylhydrazine as a chromogen [28]. For the analysis of vitamin C, the Roe's method [29] was used. Essentially, plasma specimen was diluted with metaphosphoric acid to produce a protein-free filtrate which was then stored at −20°C. Analysis was done in batches within 2 weeks after sample collection as that is the maximum period for which ascorbate stability is guaranteed at that storage temperature [30]. In a recent study in Nigeria, 0.5 mg/dL was the lower limit of normal for plasma ascorbic acid [31] and this value was used as the cutoff for vitamin C deficiency in this study.

### 2.3. Statistical Analysis

SPSS Statistical Software Package (SPSS version 11.0, SPSS Inc. Chicago, Ill, USA) was used for the data entry. Results are expressed as means ± SD. Means were compared using student's *t*-test while the categorical variables were compared using the Pearson Chi squared tests. *Z*-scores of the anthropometric measurements were calculated using the Epi lnfo statistical software package (EPI INFO version 7.0. CDC, 2011) based on WHO Child Growth Standards and WHO Reference 2007. *P* values <0.05 were regarded as significant. Pearson correlation analysis was also done to determine significant direct or inverse relationships between continuous variables. Linear regression analysis was subsequently done to identify predictors of micronutrient status among social class, body mass index, clinical stage, and immunological stages while controlling for age, gender, and albumin levels which are possible confounders.

## 3. Results

### 3.1. The Study Population and General Sociodemographics

Over the period of study, there were a total of 2057 new cases seen at the clinics of the paediatric consultant outpatient department. A hundred and nine (5.3%) of them were HIV infected. Thirty nine of the HIV infected patients were excluded from the study mainly on grounds of acute illness at presentation (25), age less than eighteen months (11), and HAART usage (3). The study population therefore comprised 70 HIV infected children (subjects) as well as 70 HIV negative children who served as controls. The age range of the subjects and control was the same (24 to 180 months). The gender ratio was similar in both subjects and control (M : F = 1 : 1.3; *P* = 0.87).  Table 1 shows the demographic, clinical, and micronutrient data of the studied subjects and controls. There were more of the infected children in the lower socioeconomic class compared to the controls and the difference was statistically significant (*P* = 0.005).

### 3.2. Nutritional Status and HIV Infection Stages

More of the infected children were stunted, wasted, and underweight compared to the controls (*P* = 0.001) (Table 1). Forty (57.1%) of the infected subjects were hypoalbuminaemic compared to 7 (14.9%) of the control (*P* = 0.001).  Table 1 also demonstrates the distribution of the HIV infected patients according to their WHO clinical and immunological stages. Forty seven (67.1%) subjects were in the late clinical stages (stages 3 and 4) while 51 (72.9%) presented with remarkable immunosuppression (severe and advanced).

### 3.3. Serum Micronutrients

Table 1 shows that the differences between the mean zinc, selenium, and vitamin C levels of subjects and the corresponding values for the controls were all statistically significant (*P* = 0.001). The percentage of HIV infected children who were deficient in each of the micronutrients was greater when compared to the controls. These differences were statistically significant (*P* = 0.001). Among the HIV infected subjects, 41 (58.6%) were deficient in all of the three micronutrients while 49 (70%) were deficient in both zinc and selenium.

### 3.4. Relationship between Serum Micronutrient Levels and WHO Clinical Stages, Immunological Categories, Nutritional Status, and Serum Albumin

**Figure 1:**
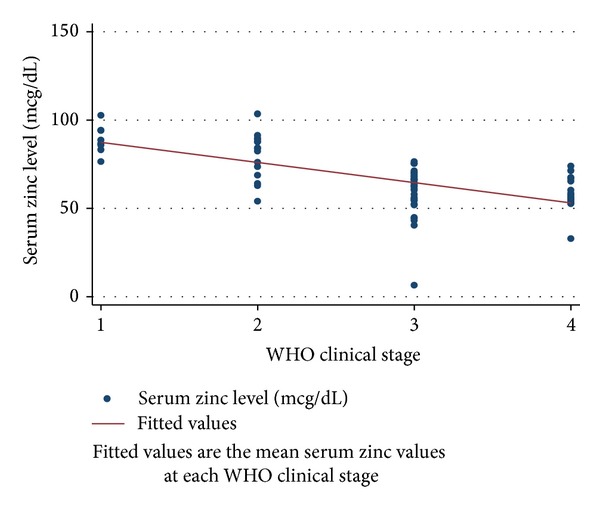
The relationship between Serum zinc levels and clinical stage of HIV infected subjects.

**Figure 2:**
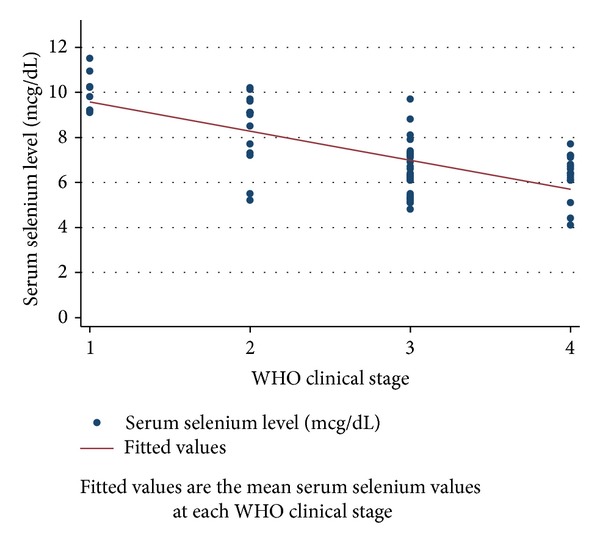
The relationship between serum selenium levels and clinical stage of HIV infected subjects.

**Figure 3:**
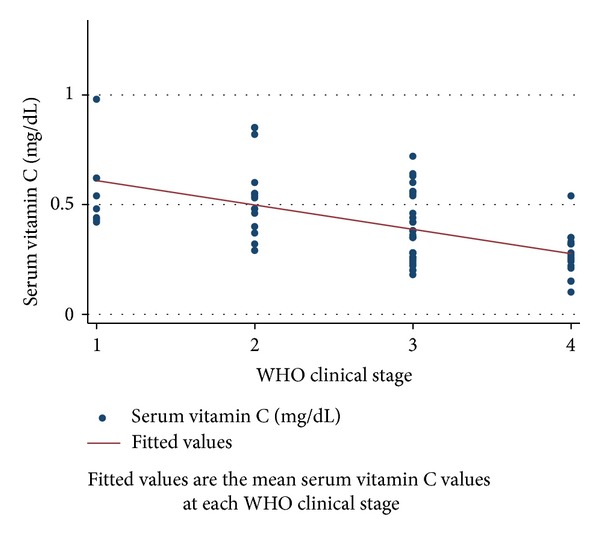
The relationship between serum vitamin C levels and clinical stage of HIV infected subjects.

Figures 1, 2, and 3 depict the progressive decrease in serum zinc, selenium, and vitamin C level with deteriorating clinical stage.  Table 2 shows the mean serum micronutrient level at various categories of immunosuppression. With both of the categorization methods employed, the mean serum micronutrient levels between early and late categories were significantly different across the three micronutrients. The weight-for-age *z*-scores were higher in HIV infected subjects who had normal serum zinc, selenium, and vitamin C levels than in those who were deficient in any of the 3 micronutrients. There was significant positive correlation between serum albumin and the serum level of each of the micronutrients (*r* = 0.386, *P* = 0.001; *r* = 0.466, *P* = 0.001; and *r* = 0.377, *P* = 0.001 for zinc, selenium, and vitamin C, resp.) (Table 3). This prompted a further linear regression analysis in which the concentration of albumin was controlled for.

### 3.5. Regression Analysis

With serum selenium, zinc, and vitamin C as the dependent variables, regression analysis was done for each of them to determine if social class, BMI, clinical, and immunological stages were predictors of each micronutrient serum levels while controlling for age, sex, and the other biochemical parameters which are potential confounders. The clinical stages as well as the advanced and severe immunosuppression categories were discovered to be significant predictors of serum selenium, serum zinc, and to a lesser extent, vitamin C. Interestingly, for all the micronutrients studied, it was found that as the clinical stages increased (to stages 3 and then 4), the regression coefficients further reduced, and the reduction was significant as well (*P* < 0.05). (Table 4).

## 4. Discussion

The significantly low levels of zinc and selenium in HIV infected subjects are similar to findings in developing countries [3, 6, 18] as well as developed countries [2, 32]. Zinc is required by HIV to form enzymes such as viral integrase, which plays a key role in its replication [6]. Prevalence of deficiency among HAART naïve HIV infected children ranges from 32% among Rwandan children [33] to 100% among Thai children [17]. Our prevalence of 77.1% falls between these. Regional disparity in the zinc intake as well as nonexclusion of children on micronutrient supplements in some of the other studies may account for the difference. Related to this higher prevalence of serum zinc deficiency in HIV infected children is the significantly lower mean of serum zinc level in HIV infected subjects as compared to the controls. In this study, the serum zinc level depreciates progressively as the disease stage worsens. This was evident in the significant direct trend between serum zinc level and increasing clinical stage (Table 4 and Figure 1) as well as between serum zinc level and degree of immunosuppression (Table 4). As the virus proliferates, the serum level of zinc reduces, perhaps due to its utilization by the virus as well as the body defence systems. A similar finding was found in other studies in children and adults [6, 17, 33, 34]. A significant percentage (57.1%) of the HIV infected patients had hypoalbuminaemia. Similar finding was also observed in Cape Town where 70% of the HAART naïve HIV infected children were hypoalbuminaemic. This is to be expected as albumin, apart from being an indicator of nutritional status, is also a negative acute phase protein [35]. In this study, albumin levels had a significant positive correlation with serum zinc level (Table 3). Albumin is known to transport at least 60% of zinc in the plasma [14]. The possibility then exists that the low serum zinc could be a result of the hypoalbuminaemia and not a true decrease in zinc level. However, when the level of albumin was controlled for in a linear regression model, the serum zinc levels particularly in clinical stages 3 and 4 as well as all levels of immunosuppression reduced significantly as the disease worsened.

The prevalence of selenium deficiency among HIV infected children in this study was 71.4% and it is higher than the range of 0% to 37% seen in literature [2, 17, 33]. The selenium level in the soil and common foods in these areas may contribute to the observed differences. Serum selenium was also demonstrated in this study, just like in some others [2, 3], to reduce in HIV infected patients as the disease worsened clinically (Table 4 and Figure 2). This is thought to result from its utilization by the body to produce glutathione peroxidase [2], an antioxidant that counteracts the prooxidant activity of HIV. This study found a statistically significant difference between mean selenium levels of infected subjects and controls (Table 1). Albumin level showed a significant positive correlation with selenium levels (Table 3). This is not surprising, as 17–32% of serum selenium is bound to albumin [14]. In this study, a linear regression model that controlled for the level of serum albumin portrayed that the serum selenium in HIV infected patients had a significant negative correlation with disease progression (Table 4).

Serum vitamin C in this study was also significantly lower in infected children than in the controls (Table 1). The prevalence of vitamin C deficiency was also significantly higher in infected subjects than in controls. These are similar to findings from other studies [9]. The antioxidant role of vitamin C in combating the prooxidant activity of HIV infection is believed to be largely responsible for vitamin C deficiency in HIV infection. In a linear regression model, serum vitamin C levels in HIV infected patients displayed a significant negative regression coefficient which reduced as the clinical stage increased (Table 3).

The prevalence of multiple-micronutrient deficiency was also similar to the observation in Cape Town [18] where 62% of the children had two or more micronutrient deficiencies. In our study, 70% of the children were deficient in both zinc and selenium with 58.6% being deficient in the three micronutrients. This knowledge will be of immense benefit as micronutrient mixtures for supplementation trial are being contemplated.

This study found HIV infected children to be generally of lower socioeconomic status compared to the HIV negative controls (Table 1). HIV prevalence is generally higher among low socioeconomic status [36]. The higher incidence among children of lower socioeconomic background has been attributed to a lower access to prevention of mother to child transmission services due to associated illiteracy, lower awareness, and poverty [37]. The observation in this study that HIV infected children had lower nutritional indices than the HIV negative controls (Table 1) has been reported in other studies [38, 39] which attributed the finding partially to poor food intake and the catabolic effects of the opportunistic infections.

It was observed that the children that had the least weight for age z-scores in this study were the HIV infected children who were also deficient in either zinc or selenium. This agrees with the Ugandan study [5] which found a positive correlation between zinc and undernutrition. A recent study found an increase of 0.3 (0.04–0.56) in mean weight-for-age z-scores among HIV infected children six months after supplementation with multiple micronutrient supplements that contained zinc [40].

We consider, as a limitation to this study, our inability to assess the dietary intake of micronutrients simultaneously with serum levels. Hence, the influence of poor intake may not be separated from the impact of the HIV itself on micronutrient status. The study being cross-sectional would also not prove causality.

In conclusion, there is prevalent deficiency in the serum levels of zinc, selenium, and vitamin C in apparently stable, ambulant, HAART naïve HIV infected children in Ile-Ife, South western Nigeria. Of significant importance is the huge percentage of multiple-micronutrient deficiency. These deficiencies are associated with low weight for age z-scores in these children. The serum levels of zinc and selenium decline as the clinical stage of HIV infection worsens with severe clinical and immunological stages being significant predictors of selenium, zinc, and vitamin C status. Socioeconomic class does not predict the serum levels of zinc, selenium, and vitamin C in HIV infected children. These findings may be a guide on the choice of the category of subjects and the outcome measures as well as the micronutrient components of supplements that would be formulated in future controlled supplementation trials in these children.

## Figures and Tables

**Table 1:** Demographic, clinical, and micronutrient data of HIV infected subjects and the HIV negative controls.

Variables	Infected [*N* = 70]	Control [*N* = 70]
Age range (mths)	24–180	24–180
Age group		
18–59 mths (*n*, %)	43 (61.4)	43 (61.4)
60–119 mths (*n*, %)	21 (30.0)	22 (31.4)
120–227 mths (*n*, %)	6 (8.6)	5 (7.1)
Mean age ± SD (mths)	58.4 ± 37.8	58.01 ± 38.1
Gender (M versus F)	40 versus 30	39 versus 31

∗Social class (*n*, %)		
I	—	9 (112.9)
II	14 (20.0)	19 (27.1)
III	16 (22.9)	15 (21.4)
^#^IV	27 (38.9)	13 (18.6)
V	13 (18.6)	14 (20.0)

Anthropometry Z scores		
^#^Median WAZ (25, 75) male	−0.18 (−0.62, 0.59)	−0.35 (−0.54, 0.38)
Median WAZ (25, 75) female	−0.53 (−0.81, 0.36)	−0.51 (−0.66, −0.15)
Median HAZ (25, 75) male	−0.13 (−0.67, 0.70)	−0.22 (0.68, 0.79)
Median HAZ (25, 75) female	−0.61 (−0.88, 0.46)	−0.53 (−0.83, 0.18)
^#^Median WHZ (25, 75) male	−1.34 (−2.29, −0.21)	−0.34 (−0.89, 0.09)
^#^Median WHZ (25, 75) female	−1.13 (−1.87, −0.44)	−0.32 (−1.11, 0.00)

^ #^Mean ± SD serum albumin (g/L)	3.2 ± 0.7	4.0 ± 0.5
^ #^Mean ± SD CD4 count (cells/mL)	611.4 ± 444.5	1388.2 ± 556.5
^ #^Mean ± SD CD4 % (%)	18.8 ± 13.2	35.4 ± 4.4

Clinical stage (*n*, %)		
1	8 (11.4)	—
2	15 (21.4)	—
3	31 (44.3)	—
4	16 (22.9)	—

Immunological stage [n (%)]		
Not significant immunosuppression	4 (5.7)	—
Mild significant immunosuppression	15 (21.4)	—
Advanced significant immunosuppression	18 (25.7)	—
Severe significant immunosuppression	33 (47.1)	—

Serum micronutrient (mean ± SD)		
^#^Zinc (*μ*g/dL)	66.99 ± 16.7	82.78 ± 25.8
^#^Vitamin C (*μ*g/dL)	0.41 ± 0.29	0.78 ± 0.28
^#^Selenium (*μ*g/dL)	7.26 ± 0.2	9.81 ± 1.6

Low albumin [*n* (%)]	40 (57.1)	7 (14.9)

Micronutrient deficiency [n (%)]		
^#^Zinc deficient	54 (77.1)	31 (44.3)
^#^Selenium deficient	50 (71.4)	13 (18.6)
^#^Vitamin C deficient	49 (70.0)	11 (15.7)

∗By Oyedeji [19]. ^#^ *P* < 0.05.

**Table 2:** Relationship between the mean serum micronutrient levels and CD4 categories of the HIV infected subjects.

	*N*	Selenium (mean ± SD)	*P*	Zinc (mean ± SD)	*P*	Vitamin C (mean ± SD)	
CD4 < 350	25	6.11 ± 1.09	0.0001	58.70 ± 16.53	0.002	0.34 ± 0.15	0.005
CD4 ≥ 350	45	7.97 ± 1.67		71.99 ± 15.07		0.46 ± 0.18	

CD4 < 25%	61	6.93 ± 1.53	0.0001	64.52 ± 16.0	0.001	0.39 ± 0.17	0.014
**CD4** ≥ 25%	9	9.52 ± 1.34		83.69 ± 11.53		0.57 ± 0.18	

*N* is the number of infected HIV subjects in that CD4 category.

*P* is significant at <0.05.

**Table 3:** Correlation of albumin with serum micronutrients level in HIV infected subjects.

	Albumin versus zinc	Albumin versus selenium	Albumin versus vitamin C
*r*	0.386	0.466	0.377
*P*	0.001	0.001	0.001
*N*	70	70	70

*N*: number of HIV infected subjects.

*r*: correlation coefficient.

*P* is significant <0.05.

**Table 4:** Predictors of serum selenium, zinc, and vitamin C in HIV infected subjects.

Predictor^#^	Selenium		Zinc		Vitamin C	
	Coefficient (CI)	*P*	Coefficient (CI)	*P*	Coefficient (CI)	*P*
Social class 2	0.91 (−0.12, 1.93)	0.08	0.30 (−9.00, 9.61)	0.95	0.07 (−0.06, 0.21)	0.28
Social class 4	0.36 (−0.61, 1.33)	0.46	6.71 (−3.19, 16.61)	0.18	0.02 (−0.11, 0.15)	0.72
Social class 3	0.73 (−0.08, 1.55)	0.08	4.71 (−4.15, 13.33)	0.29	0.06 (−0.05, 0.17)	0.25
Social class 5	0 (−1.00, 1.00)	1.00	−0.22 (−11.06, 10.63)	0.97	0 (−0.14, 0.14)	1.0
BMI	−0.82 (−0.29, 0.13)	0.43	0.09 (−2.14, 2.32)	0.08	−0.00 (−0.03, 0.02)	0.87
Clinical stage 2	−1.52 (−2.66, 0.37)	0.01∗	−11.02 (−23.33, 1.29)	0.94	−0.01 (−0.16, 0.14)	0.87
Clinical stage 3	−3.59 (−4.73, −2.45)	<0.001∗	−29.12 (−41.50, −16.74)	0.001∗	−0.18 (−0.33, −0.03)	0.02∗
Clinical stage 4	−3.70 (−5.02, −239)	<0.001∗	−30.64 (−45.85, −15.45)	0.001∗	−0.28 (−0.45, −0.10)	0.002∗
Mild immunosuppression	−0.40 (01.64, 0.84)	0.52	−1.52 (−16.31, 13.27)	0.01∗	−0.20 (−0.37, −0.02)	0.03∗
Advanced immunosuppression	−3.10 (−4.45, −1.95)	<0.001∗	−18.63 (−32.72, −4.55)	0.001∗	−0.35 (−0.51, −0.18)	0.001∗
Severe immunosuppression	−3.20 (−4.28, −1.91)	<0.001∗	−25.05 (−39.90, −10.21)	0.001∗	−0.39 (−0.56, −0.22)	0.001∗

^#^Social class 1, Clinical stage 1, and insignificant immunosuppression results were the references.

*P values were significant (*P* < 0.05).

The test of linearity and influential points proved the fit of the regression model.
